# Throughput Maximization Using Deep Complex Networks for Industrial Internet of Things

**DOI:** 10.3390/s23020951

**Published:** 2023-01-13

**Authors:** Danfeng Sun, Yanlong Xi, Abdullah Yaqot, Horst Hellbrück, Huifeng Wu

**Affiliations:** 1 Key Laboratory of Discrete Industrial Internet of Things of Zhejiang Province, Hangzhou Dianzi University, Hangzhou 310018, China; 2 Department of Electrical Engineering and Computer Science, University of Applied Science Lübeck, 23562 Lübeck, Germany

**Keywords:** spectral efficiency optimization, deep complex networks, IIoT

## Abstract

The high-density Industrial Internet of Things needs to meet the requirements of high-density device access and massive data transmission, which requires the support of multiple-input multiple-output (MIMO) antenna cognitive systems to keep high throughput. In such a system, spectral efficiency (SE) optimization based on dynamic power allocation is an effective way to enhance the network throughput as the channel quality variations significantly affect the spectral efficiency performance. Deep learning methods have illustrated the ability to efficiently solve the non-convexity of resource allocation problems induced by the channel multi-path and inter-user interference effects. However, current real-valued deep-learning-based power allocation methods have failed to utilize the representational capacity of complex-valued data as they regard the complex-valued channel data as two parts: real and imaginary data. In this paper, we propose a complex-valued power allocation network (AttCVNN) with cross-channel and in-channel attention mechanisms to improve the model performance where the former considers the relationship between cognitive users and the primary user, i.e., inter-network users, while the latter focuses on the relationship among cognitive users, i.e., intra-network users. Comparison experiments indicate that the proposed AttCVNN notably outperforms both the equal power allocation method (EPM) and the real-valued and the complex-valued fully connected network (FNN, CVFNN) and shows a better convergence rate in the training phase than the real-valued convolutional neural network (AttCNN).

## 1. Introduction

The high-density Industrial Internet of Things [1,2,3] needs to meet the requirements of multiple device access and massive data transmission, especially in fields such as augmented reality and wide-area connectivity for fleet maintenance [4,5], which requires the support of multi-antenna technology and network optimization strategies such as radio resource management. Massive multiple-input multiple-output (MIMO) technology enables the users to multiplex in the spatial domain by transmitting their signals as beams. However, the reflections in the wireless channel cause inter-user interference, turning the resource allocation problem into the non-convex (The non-convexity refers to the existence of a multitude of local maxima in the function range. It needs an exhaustive search to find the optimal solution. With such a case, systematic mathematical approaches such as the interior point method [6] are computationally too expensive to handle real-time communications.) formulation, which is hardly solvable.

In such a system, the quality of the power allocation plan will significantly affect spectral efficiency (Spectral efficiency is the normalization of the Shannon bound, which refers to the channel capacity and how many bits per second can be achieved in 1 Hz of the system bandwidth.) (SE). This motivates us to build a highly efficient power allocation plan to optimize the spectral efficiency so that we can improve the network throughput. However, the growth of the network scale and the expansion of radio resources place improving spectral efficiency and fairness processing as a crucial requirement to keep high throughput and low access latency.

Cognitive radio (CR) with multiple-input multiple-output (MIMO) systems is a potential candidate for the industrial domain [7] since CR attempts to minimize the conflict and interference between heterogeneous users creating a peaceful coexistence, as well as higher area throughput. Furthermore, in the industrial domain, the regulatory authorities of developing countries manage and coordinate the peaceful coexistence between the heterogeneous industrial networks manually. As an example, the German Federal Agency of Networks (BNetzA) has specified the band 3.7–3.8 GHz according to [8] for industrial wireless networks and imposes strict application procedures to grant licenses to the stakeholders. It is worth pointing out that such coexistence management can be automated effortlessly by means of CR technology. Therefore, the combination of massive MIMO and CR is ideal to meet the high throughput yet massive connectivity requirements.

Regarding the power optimization theory, systematic mathematical approaches such as interior point methods [6] are computationally expensive as they take centric iterations within a complex Newton step. Besides, the solution quality of these methods highly depends on the initial guess within the domain of the objective function. Heuristic algorithms are also widely used for these problems. Reference [9] used the modified lion algorithm (LA) for power allocation. The ant lion optimizer (ALO) employed in [10] achieved a good performance in fault location for power system state estimation. However, their many iterative calculations brought a great computational burden. In current real implementations, existing techniques in massive MIMO (e.g., specified in [11]) address the non-convexity issue with the equal assignment of power among users, which is obviously a sub-optimal, but time-efficient solution.

Recently, machine learning has been a hot research direction to address several wireless and networking issues [12], such as deep reinforcement learning for traffic puncturing [13], an adversarial network for adaptive antenna diagram generation [14], energy harvesting tactics [15], channel estimation of mmWaves [16], and many others.. Regarding SE optimization, Reference [17] implemented a deep neural network (DNN). The authors of [7,18] used a fully connected neural network (FNN) to estimate the best power allocation solution to maximize SE, and Lee et al. [19] proposed a convolutional neural network for power control; however, their method cannot strictly control constraints, and the FNN has the problem of unfair power allocation. Hence, Sun et al. [20] proposed the attention-based deep convolutional neural network, which has also a better time and storage space complexity. However, they all utilized real-valued neural networks to process the complex-valued channel data, which generally take the complex-valued input data as two separate parts of real-valued data. Obviously, they failed to fully take advantage of the representational capacity of complex-valued data. Furthermore, real-valued neural networks are not friendly with the non-circular complex-valued dataset (In signal processing, the complex-valued channel data are assumed circular, which is a stochastic simplification, but not always the case in reality.), as they provide less accuracy and result in more overfitting compared to the complex-valued counterparts [21].

With the advent of complex-valued neural networks (CVNNs), this problem can be well addressed. Chiheb et al. [22] proposed several key components for complex-valued deep neural networks. Reference [23] proposed complex non-parametric activation functions for CVNNs. Reference [21] implemented a tensorflow-based python library, which enabled the training and implementation of CVNNs. Yihong et al. [24] generalized meta-learning and an attention mechanism to the complex domain for signal recognition. Reference [25] proposed a sparse CVNN to acquire the downlink channel state information in the frequency division duplexing massive MIMO system.

To the best of our knowledge, no current techniques have ever applied complex-valued neural networks on power allocation for maximizing SE. Therefore, this paper proposes a complex-valued power allocation network with a complex attention mechanism (AttCVNN) to accomplish this task. Please note that the focus of this contribution is confined to the neural network design, not the beamforming processing. In more detail, our contributions are summarized as follows:

1. We propose a complex-valued convolutional neural network with a complex attention mechanism (AttCVNN) to implement the per-antenna power allocation task in massive MIMO systems.
2. Complex-valued attention mechanisms are implemented in our model, which are the complex cross-channel attention network and the complex in-channel attention network, where the former considers the relationship between cognitive users and the primary user, while the latter focuses on the relationship among cognitive users.
3. Four power allocation benchmarks are implemented to show the superiority of our model. They are the equal power allocation method (EPM), the real-valued fully connected network (FNN) [7], the complex-valued fully connected network (CVFNN), and the real-valued convolutional network (AttCNN) [20].

## 2. System Model

**Figure 1:**
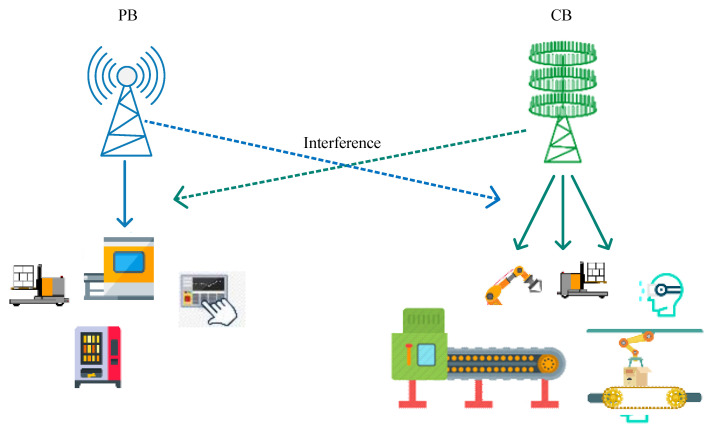
System model.

We assumed a system model, illustrated in Figure 1, that has a cognitive radio base station (CB) of *N* antennas coexisting with a primary radio base station (PB) with a single antenna. The CB communicates with *K* cognitive users (CUs) via $h_{k}\in C^{1\times N}$, where k∈[1,K], and interferes with the primary user (PU) via $h_{0}\in C^{1\times N}$. The PB communicates with a single PU via $g_{0}$ and interferes with CU *k* through $g_{k}$, where k∈[1,K].

Based on the system model, our target is to optimize the SE of the CB via a low-complexity power assignment design, which is crucial for massive connectivity applications. We formulated the optimization problem as maximizing the summation of all single CU’s SE, which must meet two constraints: C1 is used to limit the CUs consumed sum-power under the power budget of the CB ($P_{T}$), and C2 controls the actual interference $I_{CB}$ under the interference limit at the PU ($I_{th}$). $P_{PB}$ denotes the power budget of the PB. Then, the issue of SE optimization is formulated as follows.

(1) $$\begin{array}{ccc} \;\;\;\;\mathrm{SE}\;\; & = & \;\;\;\;\;\;\max_{\left\{P_{k,i}\forall k,i\right\}}\;\sum_{k=1}^{K}\;\log_{2}\;\left(\;\;1\;+\;\frac{{∥\sum_{i=1}^{N}h_{k,i}P_{k,i}^{\frac{1}{2}}∥}^{2}}{\sigma^{2}\;+\;\sum_{l\neq k}{∥\sum_{i=1}^{N}h_{k,i}P_{l,i}^{\frac{1}{2}}∥}^{2}\;+\;{∥g_{k}∥}^{2}P_{PB}\;}\right)\\ s.t.C1\;\;\;\;\;\; & : & \;\;\;\;\sum_{k=1}^{K}\sum_{i=1}^{N}P_{k,i}\leq P_{T}\\ C2\;\;\;\;\;\; & : & \;\;\;\;I_{CB}=\sum_{k=1}^{K}{∥\sum_{i=1}^{N}h_{0,i}P_{k,i}^{\frac{1}{2}}∥}^{2}\leq I_{th} \end{array}$$

where $\sigma^{2}$ denotes the Gaussian white noise variance which is the noise power, ${∥g_{k}∥}^{2}\;P_{PB}$ is the interference from PB to CU *k*, and $∥.∥$ denotes the 2-norm. $P\in R^{K\times N}$ is the power allocation solution which collects the power of *K* CUs distributed spatially over *N* transmit antennas.

## 3. Mathematical Basis for Complex-Valued Network

Compared to real-valued neural networks, a typical complex-valued neural network should possess the ability to process complex-valued inputs, which means it would contain several complex layers, such as complex dense, complex convolution, complex dropout, complex batch normalization, and others, besides that the complex-valued activation functions should also be supported.

### 3.1. Complex Convolution

For the complex-valued convolution layer with a complex-valued convolution kernel $K=K_{r}+jK_{i}$ and a complex-valued input matrix $X\;=\;X_{r}\;+\;jX_{i}$. The complex convolution performed on them can be defined as:

(2) $$Y_{out}\;=\;X*K\;=\;\;\left(X_{r}\;*\;K_{r}\;-\;X_{i}\;*\;K_{i}\right)\;+\;j\left(K_{r}\;*\;K_{i}\;+\;X_{i}\;*\;W_{r}\right)$$

where $Y_{out}$ denotes the output matrix. $K_{r},K_{i},X_{r}$, and $X_{j}$ are real-valued matrices. ∗ denotes the real-valued convolution.

### 3.2. Complex Dense

For the complex-valued dense layer with complex-valued weight matrix $W\;=\;W_{r}\;+\;jW_{i}$ and complex-valued bias vector $b\;=\;b_{r}\;+\;jb_{i}$, the output vector $y_{out}$ can be calculated as:

(3) $$y_{out}\;=\;Wx\;+\;b\;=\;\left(W_{r}x_{r}\;-\;W_{i}x_{i}\;+\;b_{r}\right)\;+\;j\left(W_{r}x_{i}\;+\;W_{i}x_{r}\;+\;b_{i}\right)$$

where $x\;=\;x_{r}\;+\;jx_{i}$ denotes the input vector.

### 3.3. Complex-Valued Activation Functions

A complex-valued activation function is needed to realize nonlinear transformation on the complex tensor. Many complex-valued activation functions have been proposed to process complex variables. They can be classified into two types, Type A would process the real part and the imaginary part of the complex variable z=x+jy separately, while Type B works in the phase and magnitude domain.

The following complex-valued activation functions proposed in this section will be used in our network; these are CRELU,RSigmoid, and RSoftmax. The complex variable *z* is defined as z=x+jy:

- CReLU would apply ReLU on the real and the imaginary part of *z*, respectively:
  (4) CReLU(z)=ReLU(x)+jReLU(y);
- RSigmoid would apply Sigmoid on the magnitude of *z*:
  (5) RSigmoid(z)=Sigmoid(|z|)
  where |z| denotes the magnitude of *z*;
- RSoftmax would apply Softmax on on the magnitude of *z*:
  (6) RSoftmax(z)=Softmax(|z|).

Note that the output of CRELU is a complex-valued number, while RSigmoid and RSoftmax would produce real-valued outputs. That is because the latter two are used to generate a real-valued output power *p* in our model. Section 3.4 shows that a complex-valued activation function does not need to satisfy the Cauchy–Riemann equation, so a complex-valued neural network utilizing the above-mentioned activation functions can be trained properly in the complex domain.

### 3.4. Complex Backpropagation

Before the backpropagation phase, a loss function needs to be defined so that we can calculate the gradient on each parameter in the network. Although the loss function takes complex numbers as the input, the output of it must be real-valued, as complex numbers are not comparable. This fact means a real-valued complex loss function is non-analytic, so we must find another way to perform a complex derivation on it. Using *Wirtinger calculus* [26], we can calculate the complex gradient for non-holomorphic functions.

The main idea of it is considering the complex function f(z) as a function of *z* and $z^{*}$, denoted as $f(z,z^{*})$, where $z^{*}\;=\;x\;-\;jy$ is the complex conjugate of z=x+jy. If *f* is real-differentiable, then $f(z,z^{*})$ will be analytic with respect to *z* when taking $z^{*}$ as constant and vice versa [27]. Thus, we can define the following partial derivatives:

(7) $$\frac{\partial f}{\partial z}\;≜\;\frac{1}{2}\left(\;\frac{\partial f}{\partial x}\;-\;j\frac{\partial f}{\partial y}\right)$$

(8) $$\frac{\partial f}{\partial z^{*}}\;≜\;\frac{1}{2}\left(\;\frac{\partial f}{\partial x}\;+\;j\frac{\partial f}{\partial y}\right)$$

We can define the complex gradient of *f* by the two partial derivatives [28]:

(9) $$\nabla_{z}f\;=\;2\frac{\partial f}{\partial z^{*}}$$

The chain rule of the loss function *L* composition with the other complex function g(z)=r(z)+js(z) can be calculated as:

(10) $$\frac{\partial L\circ g}{\partial z^{*}}\;=\;\frac{\partial L}{\partial r}\;\frac{\partial r}{\partial z^{*}}\;+\;\frac{\partial L}{\partial s}\;\frac{\partial s}{\partial z^{*}}$$

Therefore, we can train the complex-valued neural network using the equations above.

## 4. Attention-Based Complex Neural Network

**Figure 2:**
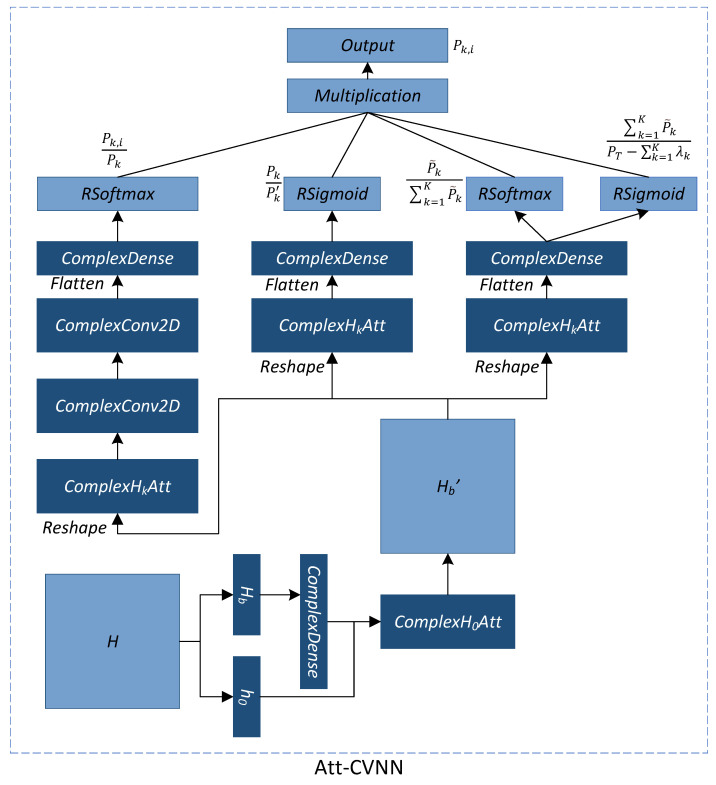
The structure of the complex-valued power allocation neural network (AttCVNN).

We propose a complex-valued convolutional neural network with an attention mechanism for the above-mentioned SE optimization problem, i.e., the AttCVNN. The AttCVNN directly takes complex-valued channel data as the input, taking complex-valued network layers as its building blocks, using complex cross-channel and in-channel attention mechanisms, i.e., the complex cross-channel attention network and the complex in-channel attention network, to improve model performance. As shown in Figure 2, the AttCVNN has a proposed data process network and three sub-networks; by multiplying the outputs of each sub-networks, we will finally obtain the allocated power for each CB user per antenna. To support complex inputs, the AttCVNN not only extends each layer to the complex domain, but realizes complex-valued attention layers, which are $ComplexH_{0}Att$ and $ComplexH_{k}Att$.

The input data are the channel coefficients, denoted as $H=[{\left[h_{1},g_{1}\right]}^{T},{\left[h_{2},g_{2}\right]}^{T},\ldots ,{\left[h_{K},g_{K}\right]}^{T},$${\left[h_{0},g_{0}\right]}^{T}{]}^{T}$, and $H_{b}={\left[h_{1}^{T},h_{2}^{T},\ldots ,h_{K}^{T}\right]}^{T}$.

### 4.1. Complex-Valued Attention

The attention mechanism is a technique that mimics the cognitive attention of human beings, which is widely used in computer vision, natural language processing, and other fields in deep learning. This mechanism would generate a weight matrix from the input data, which can be used to strengthen some parts of the input data while weakening others, making the network concentrate more on the minute, but crucial details of the data.

To employ this technique in our network, we need to extend it to support complex-valued data. Given the input matrix *X*, we can compute matrices Q, K, and V by linear transformations, which are generally implemented as fully connected layers in neural networks. The real-valued attention can be written as [29]:

(11) $$R_{Att}\left(Q,\;K,\;V\right)\;=\;Softmax\left(\frac{QK^{T}}{\sqrt{d_{k}}}\right)\;V$$

where Softmax(·) takes the cross product of Q and K as the input and, then, acts on each row of the matrix ${\mathrm{QK}}^{T}$. $d_{k}$ is a scaling factor, which denotes the row dimension of K.

For a complex-valued matrix Z, we can use a complex linear transformation to obtain complex-valued matrices $Q_{z}$, $K_{z}$, and $V_{z}$. RSoftmax is introduced to map the complex-valued matrix $Q_{z}K_{z}^{T}$ to the real domain. Then, the complex-valued attention can be written as:

(12) $$C_{Att}\left(Q_{z},\;K_{z},\;V_{z}\right)\;=\;RSoftmax\left(\frac{Q_{z}K_{z}^{T}}{\sqrt{d_{k}}}\right)\;V_{z}$$

where RSoftmax(·) takes a complex-valued matrix as the input and generates a real-valued weight matrix, which is defined in Section 3.3.

#### 4.1.1. Complex Cross-Channel Attention Network

**Figure 3:**
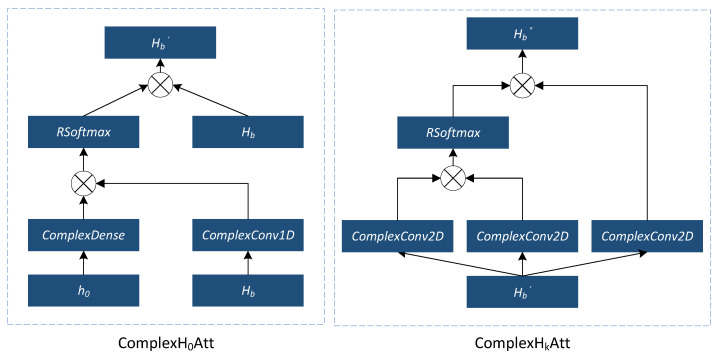
The structure of the complex-valued attention network (CVATT).

The complex cross-channel attention network, i.e., $ComplexH_{0}Att$, is designed to pay more attention to $h_{0}$, since it is strongly related to C2 and has not yet appeared in the loss function. As shown in Figure 3, the inputs $h_{0}$ and $H_{b}$ are, respectively, fed into a complex dense layer and a complex Conv1D layer. Their cross product with a complex Softmax operation cross products back to $H_{b}$ as a new $H_{b}^{\prime }$. Here, the complex dense layer is a fully connected layer, and the complex Conv1D layer is a 1D convolutional layer.

#### 4.1.2. Complex In-Channel Attention Network

The complex in-channel attention network, i.e., $ComplexH_{k}Att$, focuses on the relationship with $h_{k}$, because the definition of SE shows that the channel gain relationship among users also influences the result of SE. The input $H_{b}^{\prime }$ is fed into three complex Conv2D layers, respectively, to generate $Q_{H_{b}^{\prime }}$, $K_{H_{b}^{\prime }}$, and $V_{H_{b}^{\prime }}$. The cross product between $Q_{H_{b}^{\prime }}$ and $K_{H_{b}^{\prime }}$ would be fed into RSoftmax. Then, $H_{b}^{\prime \prime }$ is calculated by the cross product between the value of RSoftmax and $V_{H_{b}^{\prime }}$.

### 4.2. Power Allocation

The AttCVNN obtains the channel gain matrix H as the input, which will be separated into two parts, $h_{0}$ and $H_{b}$. $H_{b}$ will be fed into a complex dense layer to be preprocessed before calculating the relationship with $h_{0}$, then the two parts are fed into $ComplexH_{0}Att$ to generate $H_{b}^{\prime }$. After this, the rest of the networks are split into three parts, each of them containing a $ComplexH_{k}Att$ as their first layer and $H_{b}^{\prime }$ as their input. Their last layer is the activation functions, which will map complex-valued outputs into real values, so that their outputs can represent meaningful physical quantities. Finally, it produces $N_{1}$, $N_{2}$, $N_{3}$, and $N_{4}$ after the operations of RSoftmax, RSigmoid, RSoftmax, and RSigmoid, respectively. Considering the result range of the four operations, the outputs $N_{1}$, $N_{2}$, $N_{3}$, and $N_{4}$ can be represented as:

(13) $$\begin{array}{c} \left\{\begin{array}{c} N_{1}=\frac{P_{k,i}}{P_{k}}\\ N_{2}=\frac{P_{k}}{P_{k}^{{}^{\prime }}}\\ N_{3}=\frac{{\tilde{P}}_{k}}{\sum_{k=1}^{K}{\tilde{P}}_{k}}\\ N_{4}=\frac{\sum_{k=1}^{K}{\tilde{P}}_{k}}{P_{T}-\sum_{k=1}^{K}\lambda_{k}} \end{array}\right. \end{array}$$

where $\lambda_{k}$ means a user’s minimum power and $P_{k}^{{}^{\prime }}=\lambda_{k}+{\tilde{P}}_{k}$.

Hence, the allocated power of the *i*th antenna serving the *k* CUs can be obtained as:

(14) $$P_{k,i}=N_{1}*N_{2}*\left[\lambda_{k}+N_{1}*N_{2}*\left(P_{T}-\sum_{k=1}^{K}\lambda_{k}\right)\right]$$

Then, we build the loss function to optimize the neural network parameters as follows.

$$\begin{array}{ccc} \;\;\;\;L\;\; & = & \;\;\;\;\;\;-\;\sum_{k=1}^{K}\;\log_{2}\;\left(\;\;1\;+\;\frac{{∥\sum_{i=1}^{N}h_{k,i}{\hat{P}}_{k,i}^{\frac{1}{2}}∥}^{2}}{\sigma^{2}\;+\;\sum_{l\neq k}{∥\sum_{i=1}^{N}h_{k,i}{\hat{P}}_{l,i}^{\frac{1}{2}}∥}^{2}\;+\;{∥g_{k}∥}^{2}P_{PB}\;}\right) \end{array}$$

where ${\hat{P}}_{k,i}=P_{k,i}/\left({\left[I_{CB}/I_{th}-1\right]}^{+}+1\right)$ to meet C2.

## 5. Evaluation

### 5.1. Assessment Metric and System Configuration

The employed evaluation metric in this article is the spectral efficiency mentioned in (1) as SE. This metric corresponds to the objective of the optimization, which is the major demand in augmented reality and machine vision scenarios and applications.

We define a channel model on the basis of [30] that takes the path loss and Rayleigh fading into consideration. Regarding the model configuration, we set the path loss exponent as 2.5 and treated the distance between the CUs/PU and CB/PB as a random variable uniformly distributed ranging in $\left[10,200\right]$. The dataset contains the channel blocks. Specifically, the training examples have 1000 H’s, while the test set is 10% of the training set, where $H\in C^{10\times 100}$ and $H_{b}\in C^{9\times 99}$. Note that *K* was set to 9 in this contribution as the purpose was just to prove the concept. Then, 100 Monte Carlo realizations were performed, and the simulation curves thereof were averaged. Noise is generated as a random variable following a complex Gaussian distribution with zero mean and $\sigma^{2}=1\times 10^{-9}$, where $\sigma^{2}$ collects thermal and ambient noises. The parameters of the neural network are configured as follows: epoch =150, batch size =100, and learning rate $=1.5\times 10^{-4}$. Then, five benchmarks were built, namely, EPM, FNN, CVFNN, AttCNN, and AttCVNN. To compare the performance, SNR${}_{CB}$, SNR${}_{PB}$, and INR are defined as follows: SNR${}_{CB}=\frac{P_{T}}{\sigma^{2}}$, SNR${}_{PB}=\frac{P_{PB}}{\sigma^{2}}$, and INR $=\frac{I_{th}}{\sigma^{2}}$:

1. The EPM treats each CB user equally, and the allocated power ${\hat{P}}_{k,i}$ of the EPM is calculated as follows:
  (15) $$\begin{array}{c} {\hat{P}}_{k,i}=\frac{P_{k,i}}{{\left[K{\hat{P}}_{k,i}{∥\sum_{i=1}^{N}h_{0,i}∥}^{2}/I_{th}-1\right]}^{+}+1} \end{array}$$
  where $P_{k,i}=\frac{P_{T}}{N}$.
2. The FNN is a real-valued fully connected power allocation network, which was proposed in [7].
3. The CVFNN uses the complex dense layers as its building blocks. The input data are directly fed into three consecutive complex dense layers, then the output will be flattened and fed into four complex dense layers with the complex activation functions: RSoftmax, RSigmoid, RSoftmax, and RSigmoid, respectively, to generate the final result.
4. The AttCNN is a real-valued attention-based power allocation network, which was proposed in [20].
5. The AttCVNN is defined in Section 4, which realizes the complex-valued layers and complex-valued attention mechanism. Equations (13) and (14) are used to calculate the allocated power ${\hat{P}}_{k,i}$.

### 5.2. Training Performance for AttCVNN and AttCNN

**Figure 4:**
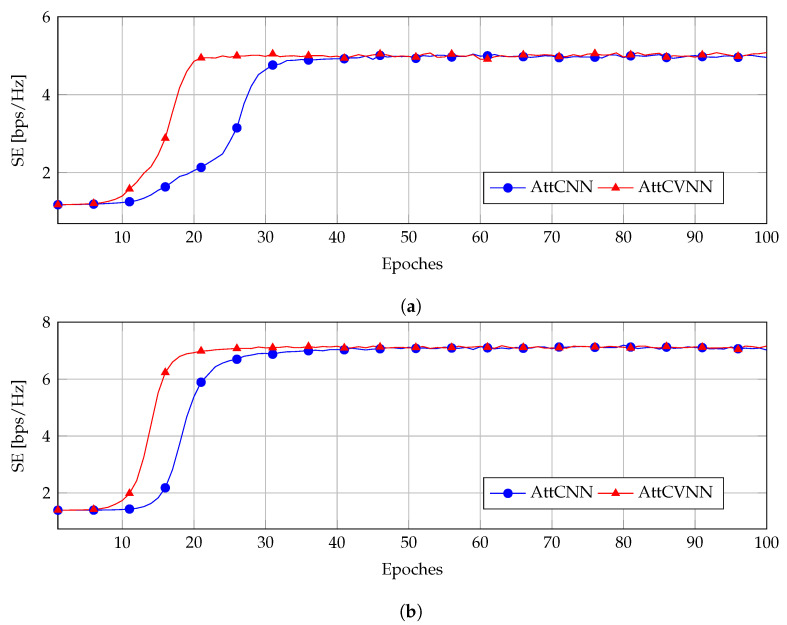
Convergence rate for the configuration INR=30 dB: (**a**) SNR${}_{PB}=60$ dB; (**b**) SNR${}_{PB}=50$ dB.

**Figure 5:**
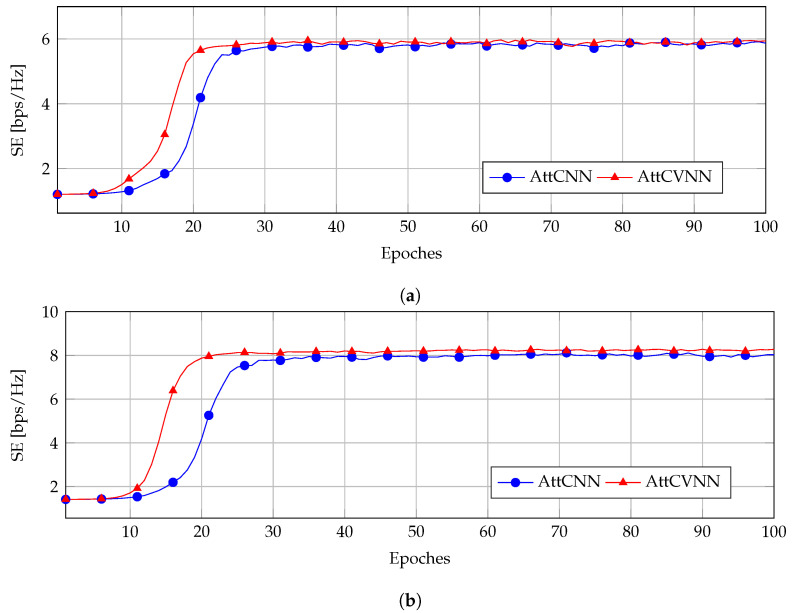
Convergence rate for the configuration INR=40 dB: (**a**) SNR${}_{PB}=60$ dB; (**b**) SNR${}_{PB}=50$ dB.

Figure 4 and Figure 5 show the training curve of the AttCNN and AttCVNN with different INRs and SNR${}_{PB}$s. We fixed the noise to $\sigma^{2}=1\times 10^{-9}$ W and the CB transmit power to $P_{T}=10$ mW. This resulted in SNR${}_{CB}=70$ dB. The interference threshold Ith was set as $1\times 10^{-3}$ mW and $1\times 10^{-2}$ mW, which correspond to an INR equal to 30 dB and 40 dB, respectively.

Throughout the experiments, although their SE curves converged to a similar value eventually, the AttCVNN has a faster convergence rate than the AttCNN. In Figure 4a,b, the SE curves of the AttCVNN reach the steady states 5 bps/Hz and 7 bps/Hz, respectively, at Epoch 20, while the AttCNN needs around 35 epochs to reach it. Figure 5a,b illustrate a similar convergence behavior, but at a higher INR setting, which relaxes the constraint *C2* and allows the SE to attain larger values, i.e., 6 bps/Hz and 8 bps/Hz, respectively.

The comparison results show that the proposed AttCVNN has a faster convergence rate than the AttCNN in the training stage, which is an advantage in real-time communications. In terms of the model design, our model holds a similar structure as the AttCNN scheme. Regarding the layer size inflation, the complex-valued implementation doubles the number of layer parameters for the sake of a rapid convergence.

### 5.3. Power Allocation Performance

We conducted two sets of comparative experiments, using the AttCVNN, EPM, FNN, CVFNN, and AttCNN, to make a comparison of their power allocation performance, where the SNR${}_{CB}$ and INR would vary from 20 to 50 dB to compare their SE performances. We assumed that $\sigma^{2}=1\times 10^{-9}$, $\lambda_{k}=0$.

#### 5.3.1. SE against SNR${}_{CB}$

**Figure 6:**
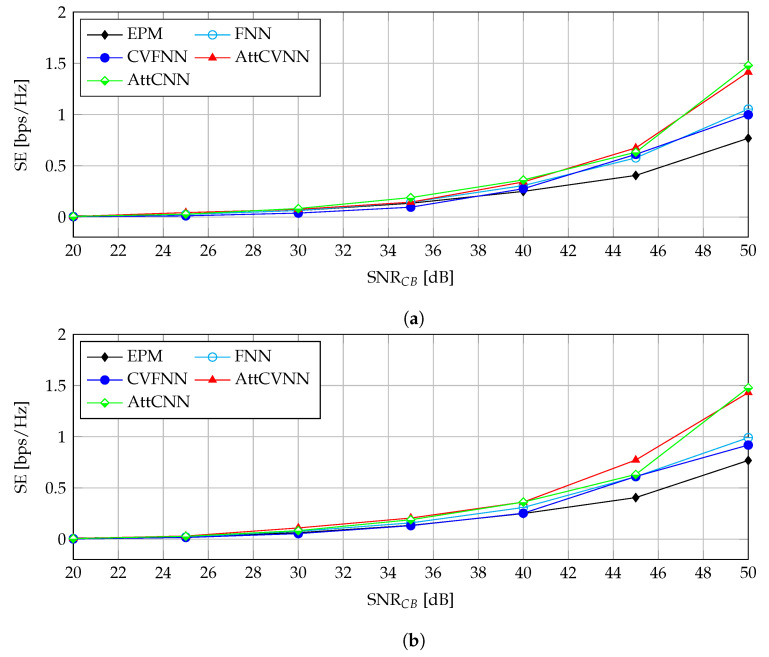
SE versus SNR${}_{CR}$ for the configuration SNR${}_{PB}=60$ dB: (**a**) INR=20 dB; (**b**) INR=30 dB.

Figure 6 demonstrates the SE against SNR${}_{CB}$ with different INRs. In Figure 6a, we set SNR${}_{PB}=60$ dB and INR=20 dB. The EPM has the lowest SE since it allocates the power equally among CB users without being able to relax the constraints and the limitations, so that the entire power budget is fully distributed among CB users. The SE performance becomes better when introducing the FNN, CVFNN, AttCNN, and AttCVNN, which use the channel knowledge H as the input to allocate and optimize the power assignment to the CB users. When the SNR${}_{CB}$ keeps increasing, the SE increases monotonically at the same time. Furthermore, the proposed AttCVNN always outperforms the EPM, FNN, and CVFNN. Note that the gap between the AttCVNN and EPM became obvious when SNR${}_{CB}=40$ dB. The gap reached almost 0.7 bps/Hz at SNR${}_{CB}=50$ dB. The AttCVNN and AttCNN have almost identical performance when the SNR${}_{CB}$ varies from 20–40 dB. However, our proposed AttCVNN is superior from the convergence rate perspective, as revealed in the previous experiments.

In Figure 6b, we set the INR at a higher value 30 dB. All curves trend monotonically, and the AttCVNN still has the best performance. Note that the EPM begins to diverge from the AttCVNN at SNR${}_{CB}=35$ dB. The gap reached almost 0.7 bps/Hz at SNR${}_{CB}=50$ dB. The AttCVNN breaks the limitation of the FNN and CVFNN with an improvement of 0.5 bps/Hz. Like Figure 6a, for SNR${}_{CB}<40$ dB, the trends of the AttCVNN and AttCNN are quite similar from the SE perspective. At SNR${}_{CB}=45$ dB, the AttCVNN outperforms the AttCNN with an SNR${}_{CB}$ gain of 2 dB. This refers to a 37% reduction in the transmit power enabled by our approach.

#### 5.3.2. SE against INR

**Figure 7:**
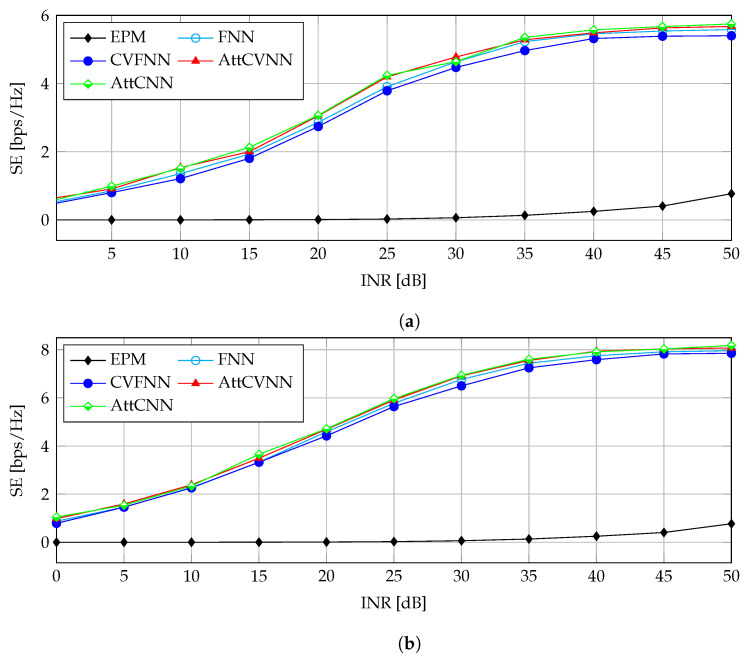
SE versus INR for the configuration SNR${}_{CB}=70$ dB: (**a**) SNR${}_{PB}=60$ dB; (**b**) SNR${}_{PB}=50$ dB.

Figure 7 introduces the results of the SE versus the INR in the range between 0 and 70 dB. The transmit power of the CB was set to $P_{T}=10$ mW, which is equivalent to SNR${}_{CB}=70$ dB. Figure 7a illustrates the SE performance for SNR${}_{PB}=60$ dB. It shows that the CR network spectral efficiency becomes high at a large INR, referring to a more relaxed upper bound for the constraint C2. In other words, the SE of the CR network increases monotonically with relaxed interference thresholds. It is worth noting that the proposed AttCVNN has always greater performance than the FNN and the CVFNN and even outperforms the EPM with a remarkable gain, e.g., 0.571 bps/Hz at INR=0 dB increasing all the way to 4.905 bps/Hz at INR=50 dB. This refers to a significant gain possibility with our proposal with an idle PR network. In Figure 7b, the same experiment is conducted, but for SNR${}_{PB}=50$ dB. It demonstrates that the SE becomes higher due to the lower SNR${}_{PB}$, which induces less interference at the CB users. Note that the proposed AttCVNN does not have a remarkable SE gain over the FNN and CVFNN in Figure 7, but it has significant horizontal or INR gain, which attains 5 dB. This implies the superiority of the AttCVNN in tighter interference conditions. Therefore, the AttCVNN and AttCNN are not distinguished in the SE performance, but in the convergence rate in favor of the proposed AttCVNN.

#### 5.3.3. Discussion

All the above experiments revealed the potential of the proposed model compared to the existing benchmarks. Moreover, all the neural-network-based methods are a huge improvement over the EPM scheme, since it does not employ any optimization theory; it only equally allocates power for the users without the consideration of interference among them. the FNN and CVFNN schemes have reasonable performance, however, associated with a large number of parameters, leading to severe overfitting. This limits their performance improvement. The AttCVNN and AttCNN use the convolutional layers to reduce the amount of parameters to prevent overfitting, and the introduction of the attention mechanism significantly improved their performance. However, the complex-valued implementation speeds up the process of training, which is a major advantage in real-time communications.

### 5.4. Computational Complexity

In practice, we generally use floating-point operations per second (FLOPs) to measure the time complexity of neural network models. With the experiment configuration, the time complexity of our model is 17.92 million FLOPs. As a comparison, the MobileNetV3-Small [31], proposed for mobile phone CPUs, has a time complexity of 59 million FLOPs. With more powerful processors, our model can support industrial applications with lower computational complexity.

## 6. Conclusions

This paper proposed a novel attention-based complex-valued power allocation network, the AttCVNN, to optimize the power allocation performance, where complex in-channel and cross-channel attention networks were implemented. We performed comparative experiments by varying the SNR${}_{CB}$ and INR. Compared with the designed benchmarks (i.e., EPM, FNN, CVFNN, and AttCNN), it was shown that the proposed AttCVNN outperforms the EPM, the FNN, and the CVFNN notably regarding SE. The proposed model has faster convergence than the AttCNN in the training phase, which is a major advantage in real-time communications. The AttCVNN is a promising method for enhancing the throughput performance via radio resource management and optimization in the IoT scenarios of Industry 5.0.

## Data Availability

Not applicable.
